# Mapping drug distribution using CT imaging following direct tissue injection in ex vivo liver: informing clinical implementation

**DOI:** 10.1007/s44343-025-00027-x

**Published:** 2025-12-16

**Authors:** Robert Morhard, Michal Mauda-Havakuk, Jose F. Delgado, Michael T. Kassin, Azam Ghafoor, Ira Pastan, Raffit Hassan, John W. Karanian, William F. Pritchard, Bradford J. Wood, Andrew S. Mikhail

**Affiliations:** 1 Center for Interventional Oncology, Radiology and Imaging Sciences, Clinical Center, National Institutes of Health, Bethesda, MD USA; 2 Interventional Oncology Service, Interventional Radiology, Tel Aviv Sourasky Medical Center, Tel Aviv District, Israel; 3 Thoracic and GI Malignancies Branch, Center for Cancer Resaerch, National Cancer Institute, National Institutes of Health, Bethesda, MD USA; 4 Laboratory of Molecular Biology, National Cancer Institute, National Institutes of Health, Bethesda, MD USA; 5 Thoracic and Solid Tumor Immunotherapy Section, Thoracic and GI Malignancies Branch, Clinical Center, National Institutes of Health, Bethesda, MD USA; 6 Institute of Biomedical Imaging and Bioengineering and National Cancer Institute Center, Bethesda, MD USA

**Keywords:** Intratumoral injection, Image-guided drug delivery, Locoregional therapy, Drug distribution modeling, Injection technique optimization

## Abstract

**Purpose:**

Direct intratumoral injection of therapeutic drugs can minimize total dose and adverse effects compared to systemic administration. Leakage from the injection site may cause variable drug distribution and efficacy, and off-target toxicity. This study aimed to evaluate the co-injection of an iodinated contrast agent with an imageable surrogate drug (fluorescent albumin) to estimate spatial drug distribution.

**Methods:**

Fluorescent albumin and iodixanol were injected (1, 2, or 4 mL at 1 mL/min) into ex vivo bovine liver. Distribution of iodine on CT was compared to fluorescent albumin on fluorescence microscopy, including comparison of distribution volume in liver as a function of injected volume. Physical properties (hydrodynamic diameter, zeta potential) of both iodixanol and fluorescent albumin were measured.

**Results:**

In comparison to fluorescent albumin, iodixanol is smaller (2.7 ± 0.4 nm vs. 17.0 ± 1.7 nm) and more neutrally charged (2.3 ± 0.5 mV vs. − 17.7 ± 1.4 mV). The distribution volume of iodixanol in tissue is approximately 7 times greater than that of fluorescent albumin. However, the correlation of distribution volumes of both agents is *R*^2^ = 0.89. The variance in distribution volume in tissue of iodixanol and fluorescent albumin increased with injection volume.

**Conclusion:**

The distributions of contrast and surrogate drug were correlated; however, differing physicochemical properties caused differences in their distribution. Although drug and contrast may not colocalize, contrast may serve as an imageable surrogate to inform preclinical development and clinical applications. This study suggests that dividing an interstitial injection into multifocal small-volume injections results in better localization of the injected drug at the target.

**Trial registration:**

NCT04840615.

**Supplementary Information:**

The online version contains supplementary material available at 10.1007/s44343-025-00027-x.

## Introduction

Intratumoral drug delivery by percutaneous injection into solid tumors provides advantages over systemic delivery, including reduced systemic exposure and increased intratumoral drug concentrations [1]. Local intratumoral injection may reduce non-target toxicities and side effects compared to systemic drug delivery and allow on-target effects at a fraction of the systemic dose. However, variations in injection techniques and heterogeneous tumor biophysical properties result in inconsistent and unpredictable drug distribution or leakage into surrounding tissue [2]. This may limit deposition of drug in the tumor, potentially compromising the efficacy and safety of treatment [3]. Further, the utility of image guidance for injections is incompletely understood and remains non-standardized. Broader clinical adoption of intratumoral injections would benefit from an improved understanding of the influence of injection parameters on drug distribution following interstitial injection.

One barrier to the penetration of tumors by systemically administered drugs is elevated tumor interstitial fluid pressure. High interstitial pressure reduces the pressure gradient between the microvasculature and interstitium and thus diminishes transvascular fluid flow into the extravascular tumor compartment [4]. Furthermore, since high pressure regions may exist towards the center of a tumor, drug that does extravasate into the tumor may be cleared rapidly, distribute beyond the tumor target, or remain confined to the lower pressure tumor periphery [5]. Intratumoral injections can generate fluid pressures an order of magnitude greater than the tumor interstitial fluid pressure, resulting in convective fluid flow away from the needle tip that can disperse drug throughout a tumor or beyond tumor margins [6]. However, pressurized fluid can fracture tissue creating a route for injected drug to leak directly into lower pressure regions, such as blood or lymphatic vessels and adjacent tissues, instead of distributing uniformly [7]. There are currently no standardized technical guidelines for intratumoral drug injections to inform the optimal selection of needle type and the volume and rate of injection to localize delivery and limit the effects of tissue fracture. Moreover, most drugs are not visible using clinical imaging, limiting the ability for image-guided delivery to estimate the distribution of locally administered drugs and evaluate real-time on-target delivery.

In this study, we investigated the use of a locally injected iodinated contrast agent that is imageable on computed tomography (CT) to assess the delivery of a fluorescent surrogate drug with the goal of clarifying the impact of dose fractionation (i.e., dividing the total injection volume into multifocal small injections) on the localization of an injected drug. Dose fractionation was modeled by varying the volume of a mixture of contrast agent and fluorescent albumin injected into ex vivo bovine liver. CT provided three-dimensional visualization of the injectate distribution. The objective of this study was to determine if co-injection of a commonly used CT contrast agent, iodixanol, with a fluorescent surrogate drug could enable estimation of drug tissue distribution on CT, and to assess the impact of injection volume on surrogate drug localization in liver. Fluorescent albumin was selected as a simulated surrogate drug because it is similar in size to a novel immunotoxin (LMB-100) that was being evaluated for the treatment of mesothelioma in a clinical trial (NCT04840615). However, it is larger and more negatively charged than the contrast agent iodixanol. LMB-100 is a prime candidate for intratumoral injection because systemic exposure leads to side effects such as vascular leak syndrome and the robust development of anti-drug antibodies, which may limit the efficacy of repeat treatments [8–11]. This work demonstrates the impact of dose fractionation and motivated an approach to intratumoral injection in a clinical trial. The imageable surrogate methodology applied here may potentially be extended to optimize injection parameters, needle selection, and spacing of injections, evaluate the inclusion of in situ gelling polymers, or generate technical guidelines for intratumoral injections.

## Materials and methods

### Physical characterization

Hydrodynamic diameter and zeta potential were measured with a Malvern ZetaSizer NanoSeries (Malvern Panalytical, Westborough, MA, USA). For hydrodynamic diameter measurements, fluorescent albumin (ThermoFisher, Waltham, MA, USA) and iodixanol (Visipaque, 320 mg iodine/mL, GE Healthcare, Marlborough, MA, USA) were dissolved in 5 mM saline at concentrations of 0.5 mg/mL and 5.0 mg iodine/mL, respectively. These concentrations provided an adequate signal (at least 10 kilocounts of detected scattered photons per second greater than saline alone) while minimizing the frequency of multiple scattering events. Zeta potential measurements were conducted at fluorescent albumin and iodixanol concentrations of 0.1 and 1.0 mg/mL, respectively, dissolved in 1 mM saline. These concentrations were selected to ensure the signal was discernible from the background and the scattered light was not being attenuated as advised by the manufacturer. Measurements entailed a minimum of 20 kilocounts of detected scattered photons per second, and the corresponding phase plot had a single peak, indicating that scattered light was not being attenuated. Solutions were filtered through a 0.22-µm syringe filter (Millex GS, Millipore, Rockville, MD, USA) before measurement. Five independently prepared solutions (pH 7.0–7.2) were measured using a Hach sensION + PH3 (Hach Company, Loveland, CO, USA). Each solution was measured in triplicate and the values were averaged.

### Injection and freezing procedure

Injection solutions were composed of 10 mg/mL fluorescent albumin containing 25% (w/w) FITC-labeled bovine serum albumin and 75% unlabeled bovine serum albumin (Sigma Aldrich, St. Louis, MO, USA) dissolved in water and mixed with the contrast agent iodixanol to a final iodine concentration of 32 mg I/mL. Ex vivo bovine liver (Balducci’s Market, Bethesda, MD, USA) was cut into blocks of approximately 5 × 5 × 10 cm, and sets of 3 were placed into plastic containers. The tissue was warmed to room temperature before the injection. Injection needles (21G Winged Infusion Set; Terumo, Leuven, Belgium) were inserted under CT imaging guidance with the final position selected to avoid air-filled vessels. Using a dual-syringe pump (PHD Ultra, Harvard Apparatus, Holliston, MA, USA) and a single-syringe pump (InfusionOne, New Era Pump Systems, Farmingdale, NY, USA), 1, 2, or 4 mL of the fluorescent albumin-iodixanol solution was injected at 1 mL/min. Three injections at each specified injection volume were performed simultaneously, one in each liver block. Individual injections were considered biological replicates. A total volume of 4 mL was chosen as the highest volume because this was specified to reflect the protocol in the clinical trial (NCT04840615) and was the total dose for the initial study of T-VEC that is foundational to the use of intratumoral injections [12]. Following injection of the solutions, CT images were acquired at 2 min, and samples were subsequently submerged in optimal cutting temperature (OCT) compound and placed in a − 80 °C freezer for a minimum of 3 days to allow samples to fully freeze.

### CT imaging and analysis

CT images were acquired at 120 kVp and 250 mAs with 0.8 mm thick sections reconstructed at overlapping 0.4 mm intervals (Brilliance MX8000 IDT 16-section detector CT; Philips, Andover, MA, USA). The distribution volume and radial concentration profile of the injectate were quantified with 3DSlicer (https://www.slicer.org) [13]. CT images of tissue samples were cropped to exclude contrast that leaked outside of the tissue. The tissue boundary was defined as approximately 5 mm above the bottom of the container to ensure reproducibility. The distribution volume was defined as voxels within liver tissue with a radiodensity in Hounsfield units (HU) that exceeded a manually selected threshold value of 150 HU that separated the injectate from background. A threshold of 150 HU was chosen for all samples because it was the lowest pixel value not exceeded in pre-injection imaging of the liver. The thresholding and analysis of the injectate distribution volume were automated using custom Python code. The radiodensity frequency distribution and its dependence on distance from the centroid of the injectate distribution were calculated using custom MATLAB (Mathworks, Natick, MA, USA) code.

### Fluorescent imaging and analysis

The frozen tissue blocks were imaged with a Quantum GX2 microCT (90 kV, 88 µA , 2 min scan time; PerkinElmer, Waltham, MA) to locate the injectate and guide the cutting of the blocks to a 2–3 cm thick slab. The slab of frozen tissue was sliced into 20 μm sections and 3 representative sections were cut perpendicular to the axis of the needle through the center of the injectate, mounted on glass slides, and imaged with both fluorescence (Aperio ScanScope FL, excitation = 480 nm, emission = 535 nm, exposure time = 80 ms, magnification = × 20; Leica Biosystems, Deer Park, IL, USA) and brightfield (Aperio ScanScope XT, exposure time = 10 ms, magnification = × 20; Leica Biosystems) slide scanners. Representative sections considered for analysis were spaced by 100 µm.

The distribution volume of fluorescent albumin was approximated by computing the area of the manually outlined fluorescent region and extrapolating to an effective volume, assuming a spherical geometry. This approximation was validated using CT images of the injections by calculating the area of the largest cross-sectional plane, approximating the volume, and comparing it to the total volume. The average absolute discrepancy was 29%. The fluorescence intensity as a function of distance from the center of the distribution, the frequency distribution, and the estimated concentration were calculated using custom MATLAB code. The distribution volumes in tissue of iodixanol and fluorescent albumin were determined by correlating the distribution volumes of each determined using CT and fluorescence imaging, respectively.

The fluorescent albumin concentration was estimated by fluorescence imaging of calibration standards of known concentration. Calibration standards were created by pipetting 10 µL solutions of fluorescent albumin ranging from 0.5 to 20 mg/mL (*C*_solution_, *n* = 18) onto the surface of 20 µm thick sections of bovine liver tissue. The concentration was determined using Eq. 1, in which the volume of the dried solution is quantified by multiplying the fluorescent area (*A*_fluorescence_) by the tissue thickness (*h*_tissue_). The area is determined by manual segmentation, and the fluorescent intensity was averaged under the assumption that the fluorescent intensity was uniform. The tissue concentrations (*C*_tissue_) ranged from 5 to 350 mg/mL.

$${\text{C}}_{\mathrm{solution}}{\text{V}}_{\mathrm{solution}}= {\text{C}}_{\mathrm{tissue}}{\text{V}}_{\mathrm{tissue}}$$

$${\text{C}}_\mathrm{solution}{\text{V}}_\mathrm{solution}={\text{C}}_\mathrm{tissue}{\text{A}}_\mathrm{fluorescence}{\text{h}}_\mathrm{tissue}$$

1 $$\frac{\text{C}_{\text{solution}}*10\ \upmu\text{L}}{\mathrm{A}_{\textrm{fluorscence}}*10\ \upmu\mathrm{m}}={\mathrm{C}}_{\mathrm{tissue}}$$

### Clinical implementation

Local image-guided injection of immunotherapy demonstrated the principles of this work in a Phase 1 clinical trial (Fig. 5). Written informed consent was obtained with enrollment in an Institutional Review Board approved study protocol (NCT04840615). This clinical study examined whether intratumoral injection of anti-mesothelin immunotoxin LMB-100 had greater anti-tumor effects when combined with the immunotherapy drug ipilimumab in patients with mesothelioma.

Image-guided direct injection of 4 mL of LMB-100 into the mesothelioma tumor was performed with US and PET CT fusion guidance, with registration of pre-procedural PET to procedural imaging to facilitate injection into tumor volumes with “hot” PET (i.e., high SUVs). The clinical image is reported under a retrospective review protocol approved by the Institutional Review Board that met the criteria for waiver of patient consent. The injection of 4 mL total of LMB-100 was done without the use of a radiographic contrast agent and was performed with a multipronged needle with three curved injection tines advanced from the sides of the needle (Quadra-Fuse Rex Medical, Conshohocken, PA, USA). The needle was deployed with injections at incremental sequential deployment distances of the injection tines, followed by withdrawal of the tines, 60° rotation of the base needle, and repeat staged deployment with small aliquot injections at each stage. Standardized rotation and serial staged deployments promoted broader distribution in the target tumor. CT and ultrasound guidance were used with verification of target tissue delivery via confirmation of base needle location and real-time US visualization of the tine deployment and the injection. US monitoring of the injection allowed for stopping any single site or stage injection when extra-tumoral extravasation or fracture occurred.

### Statistical analysis

Linear regression was performed between fluorescent albumin concentration and fluorescence intensity and between iodine concentration and radiodensity using the MATLAB function “fitlm”. This function was also used to compare the distribution volumes as measured by CT and fluorescence imaging. Descriptive results are reported as mean ± standard deviation. Error bars depict the standard deviation. 95% confidence intervals for the slopes of regression lines were quantified using the “fitlm” function, and for *R*^2^ coefficients using accepted formulas [14]. Statistical analysis of the hydrodynamic diameter and zeta potential of iodixanol and fluorescent albumin entailed an unpaired *t*-test. Normality was confirmed by a Shapiro–Wilk test using the MATLAB function “swtest”. To evaluate the robustness of the regression given the limited sample size, a leave-one-out analysis was performed. Each data point was sequentially excluded, the regression model was re-fit to the remaining data, and the coefficient of determination (*r*^2^) was recalculated.

## Results

### Characterizing size, charge, and imageability

**Fig. 1:**
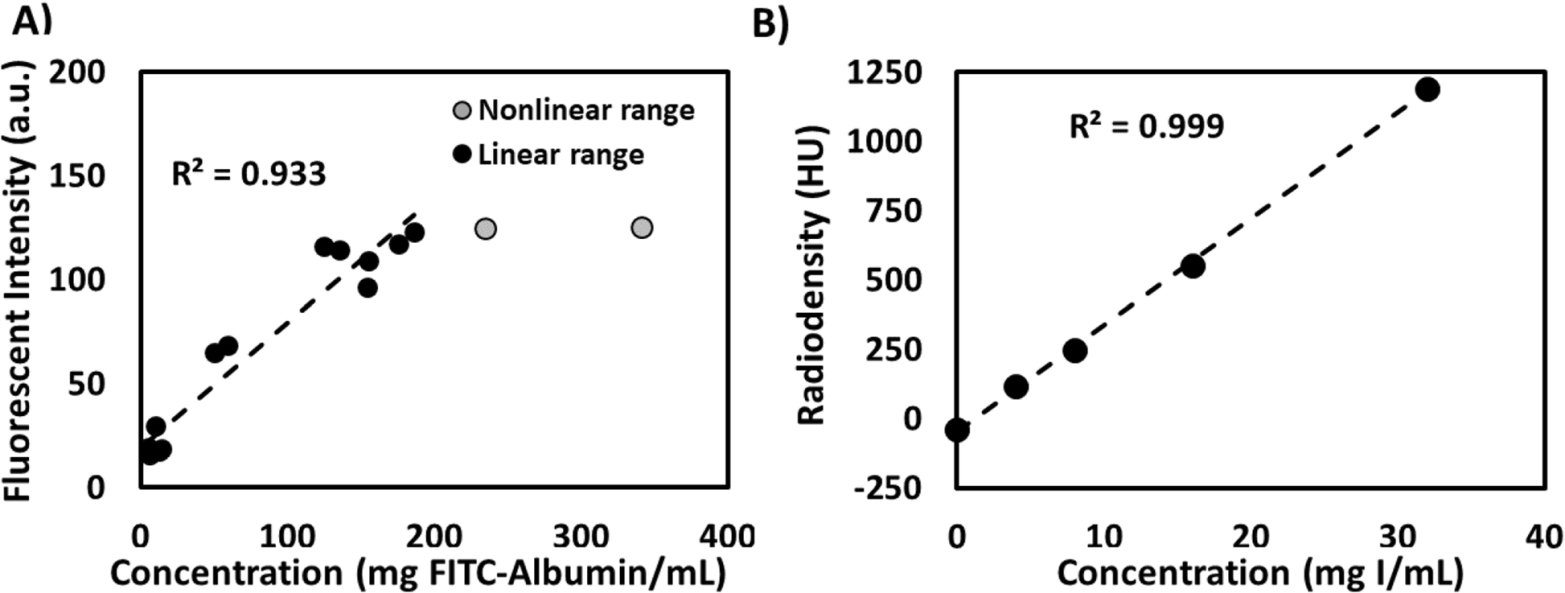
Optical and CT imaging of fluorescent albumin and iodixanol. **A** Quantifying the fluorescence intensity of calibration standards illustrates a linear region below 200 mg/mL with self-quenching behavior at greater concentrations (signified by the gray points). **B** The radiodensity of iodixanol is linearly proportional to its concentration

At a neutral pH, iodixanol had a smaller hydrodynamic diameter than fluorescent albumin (2.7 ± 0.4 nm vs 17.0 ± 1.7 nm, *p* < 0.0001). Iodixanol also had a more neutrally charged zeta potential than fluorescent albumin (2.3 ± 0.5 mV vs. − 17.7 ± 1.4 mV, *p* < 0.0001). To approximate the relationship between fluorescence intensity and fluorescent albumin concentration, calibration standards were imaged with the same imaging parameters and tissue thickness as sectioned tissue. As illustrated in Fig. 1A there was an approximately linear increase in fluorescence intensity with fluorescent albumin concentration up to a concentration of 200 mg/mL (approximately 2.5 mM based on a molar mass of 70 kDa provided by the manufacturer). The slope of the trendline in Fig. 1A is 0.60 (95% CI [0.48, 0.72]) with an R^2^ of 0.933 (95% CI [0.858, 1.000]). Self-quenching has been observed at FITC concentrations in excess of 20 mM [15], which corresponds with our findings, given albumin labeling with 12 FITC molecules ($2.5\text{ mM FITC}-\text{Albumin}$ * $\frac{12\text{ FITC}}{1\text{ Albumin}}=20 \text{ mM FITC})$. The relationship between iodine concentration and radiodensity was linear throughout the experimental range (Fig. 1B). The slope of the trendline in Fig. 1B is 39.5 (95% CI [39.1, 39.9) with an *R*^2^ of 0.999 (95% CI [0.998, 0.999]).

### CT imaging of iodixanol

**Fig. 2:**
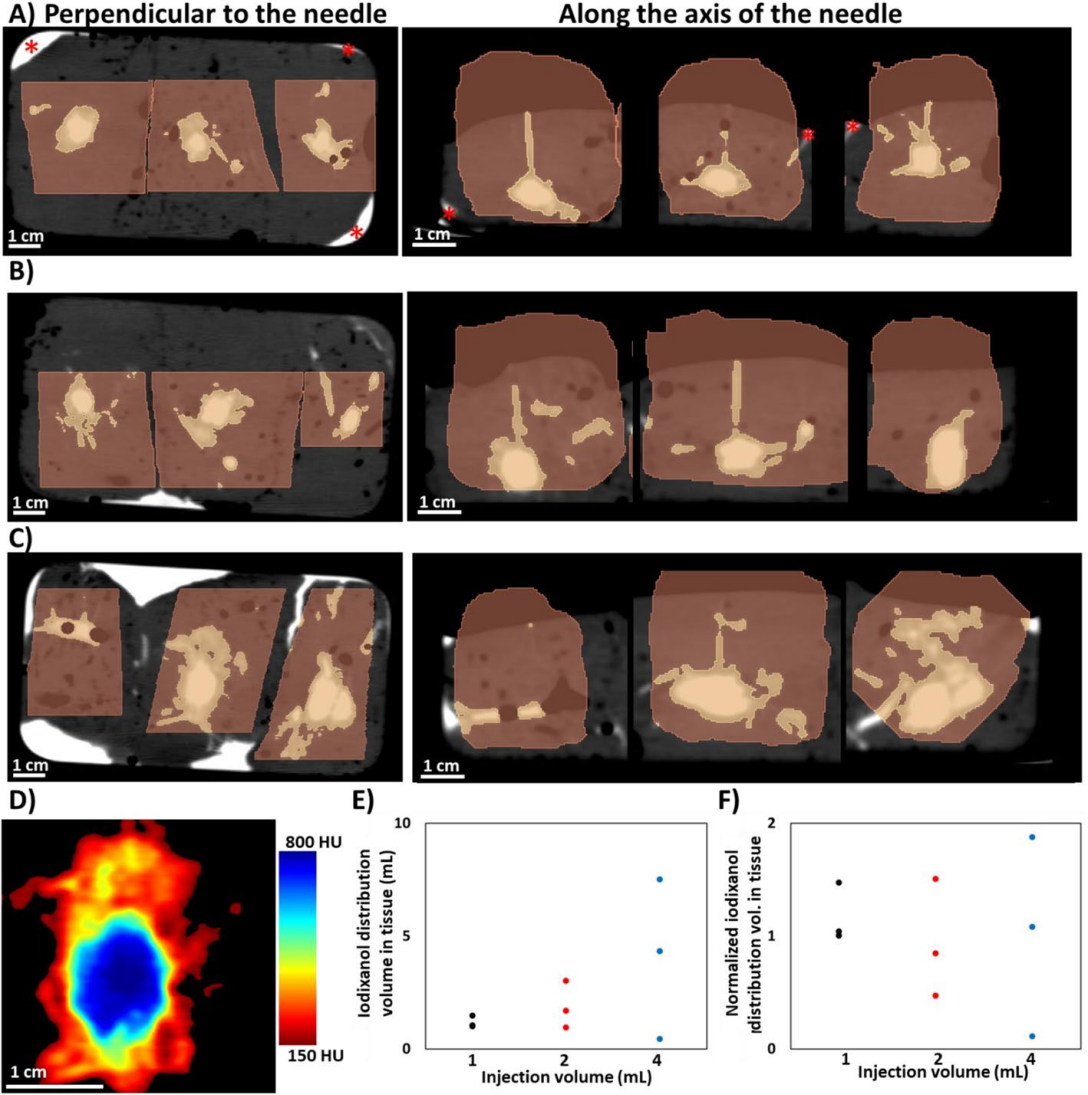
CT imaging of iodixanol distribution. CT images were acquired within 2 min of the simultaneous injection of three liver samples with injection volumes of **A** 1 mL, **B** 2 mL, and **C** 4 mL. The CT sections shown are perpendicular to the needle pathway (left hand column) and parallel to the needle axis (right hand column). The images are grayscale with liver tissue in dark gray, surrounding air in black, and injectate in white. Injectate that leaked out of the tissue and collected within the container appears white (asterisks mark several areas in A as an example). Leakage of contrast was most apparent for the 4 mL group. Segmented tissue has a brown overlay while the segmented injectate distribution, which was defined by a radiodensity threshold of 150 HU, has a yellow overlay. The brightest yellow appears in regions of high contrast concentration. Scale is consistent across all 3 sets of images. **D** A representative injection illustrates the radial dependence of the iodixanol concentration profile, displayed as a color heatmap. **E** The distribution volume of each sample was calculated from the CT images acquired immediately after the injection. **F** The average normalized distribution volume (volume in liver measured on CT/injection volume) of iodixanol is approximately 1 for all injection volumes and the variance increases with injection volume

CT imaging of co-injected iodixanol facilitated rapid and non-destructive imaging of the injectate distribution and quantification of its morphology in three dimensions. Cross-sections of each injectate distribution acquired within 2 min of 1-, 2-, and 4-mL injections are illustrated in Fig. 2A–C, respectively**.** Iodixanol leakage from the tissue was most apparent for the 4 mL group. A representative injectate distribution from a 4 mL injection (Fig. 2D) in a section perpendicular to the needle pathway demonstrates the radial dependence of the iodixanol concentration profile. Iodixanol distributed over a volume of 1.17 ± 0.26 mL, 1.88 ± 1.05 mL, and 4.09 ± 3.54 mL for 1 mL, 2 mL, and 4 mL injections (*n* = 3 each) (Fig. 2E). The normalized distribution volumes, defined as the ratio of the distribution volume to the injection volume, for 1 mL, 2 mL, and 4 mL injections were 1.17 ± 0.26, 0.94 ± 0.52, and 1.02 ± 0.88, respectively (Fig. 2F). Although the average normalized distribution volume was independent of the injection volume, the variance of the normalized distribution volume increased with injection volume.

### Imaging of fluorescent albumin

**Fig. 3:**
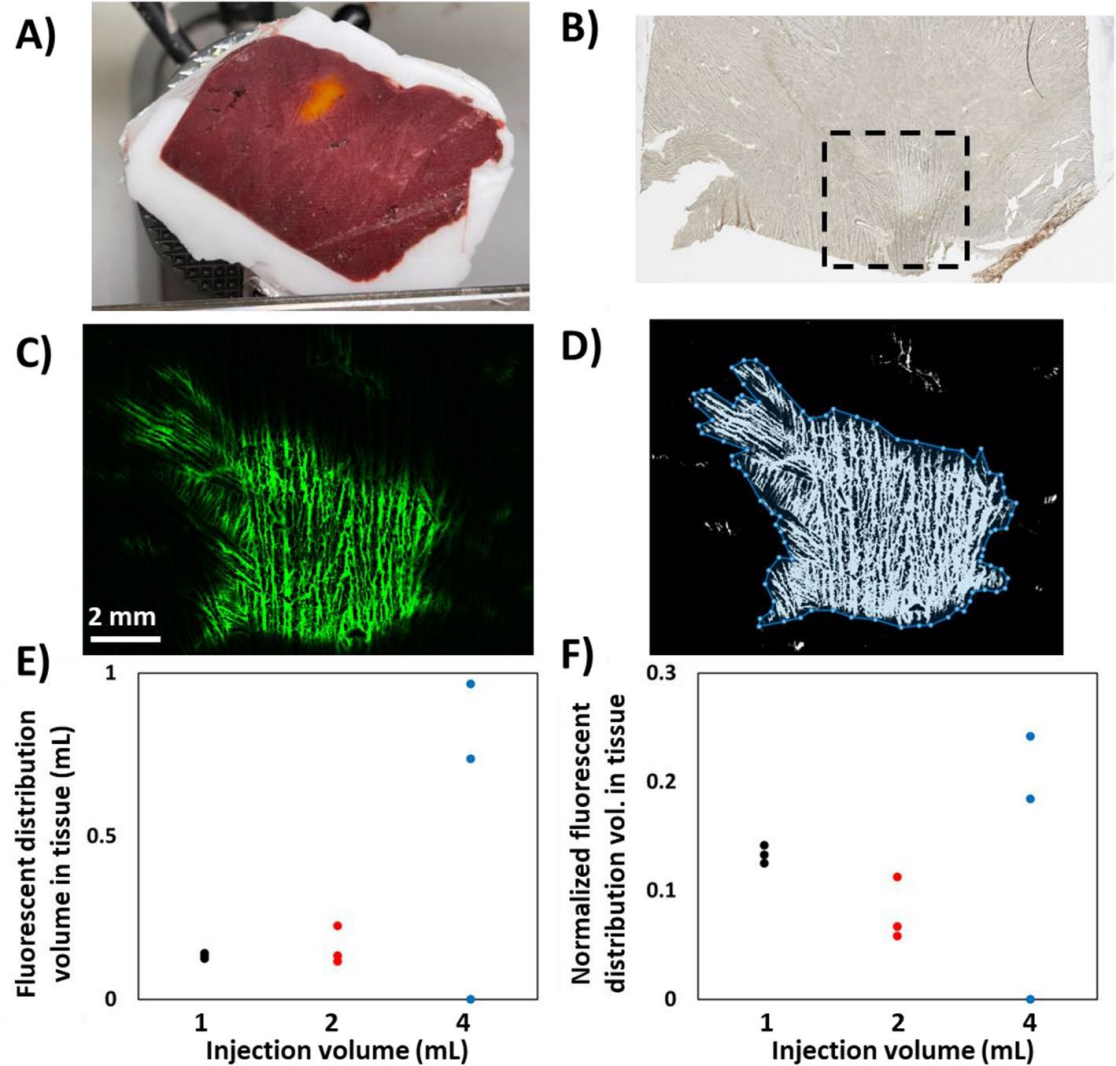
Optical imaging of fluorescent albumin distribution in liver. Samples were embedded in OCT and frozen at − 80 °C before three 20 µm sections were cut in the approximate center of the distribution with a cryotome. **A** FITC appeared orange in the tissue sample. **B** The injection site was faintly visible on a brightfield microscope image of a tissue section, and **C** was conspicuous with fluorescence microscopy. **D** To determine the area of the fluorescent region on each section, an ROI was traced around the fluorescent region of a grayscale image. **E** The average fluorescent area and an equivalent radius were calculated for each sample and used to approximate the total distribution volume. **F** The normalized distribution volume, distribution volume/injection volume, for all samples was much less than 1, demonstrating that fluorescent albumin stays relatively localized

The fluorescent albumin distribution within frozen tissue appears orange under ambient light (Fig. 3A). Tissue was embedded in OCT compound to preserve the structure during freezing and sectioning. After sectioning, brightfield images of unstained tissue were acquired to ensure there are no tears near the injectate distribution (Fig. 3B). Fluorescence images were obtained to visualize the fluorescent albumin distribution (Fig. 3C), and the fluorescent region was manually outlined using MATLAB to approximate the distribution volume (Fig. 3D). Striations in the fluorescence distribution were likely the result of ice crystal formation during freezing.

Approximated distribution volumes for each sample are plotted in Fig. 3E, with 1-, 2-, and 4-mL injections distributing over 0.14 ± 0.01 mL, 0.17 ± 0.04 mL, and 0.60 ± 0.53 mL, respectively (*n* = 3 each). In one of the 4 mL samples, the injectate resided in the void space of the vasculature and therefore could not be sectioned and transferred to a glass slide such that fluorescence images could not be captured. The normalized distribution volumes are plotted in Fig. 3F. The. 1-, 2-, and 4-mL injections have normalized distribution volumes of 0.14 ± 0.01, 0.08 ± 0.02, and 0.15 ± 0.13 mL, respectively. The average normalized distribution volume over all samples was 0.12 ± 0.07 mL. The average normalized distribution volume for fluorescent albumin was smaller than that of iodixanol (0.12 ± 0.07 vs. 1.05 ± 2.26). Similar to CT imaging of the injectate distribution, the average normalized distribution volume does not increase with injection volume, but the variance does.

### Analysis of concentration profiles and correlation of distribution volumes

**Fig. 4:**
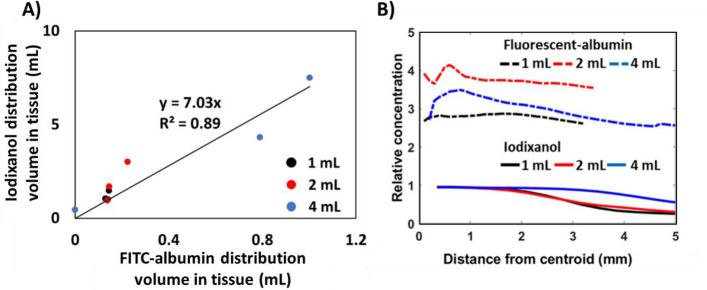
Radial and frequency distributions of iodixanol and fluorescent albumin in liver. **A** The iodixanol distributed over a larger volume than the fluorescent surrogate drug, but there was a strong correlation between the spatial distribution of the two molecules. **B** The iodixanol exhibited a strong radial dependence with a maximum concentration at the centroid approximately equal to the injection solution concentration (i.e., relative concentration = 1). The surrogate drug accumulated in highly concentrated regions (relative concentration > 1) with weaker radial dependence and sharper boundaries compared to iodixanol

An objective of this study was to assess the correspondence of a co-injected contrast agent with a fluorescent surrogate drug following interstitial injection. Figure 4A illustrates that while the distribution volumes quantified via fluorescence and CT imaging are not equivalent, they are correlated (*r*^2^ = 0.89, 95% CI [0.80, 0.98]). The distribution volume of iodixanol as determined by CT imaging was 7 times greater than that estimated for fluorescent albumin (95% CI [5.45, 8.60]). The leave-one-out analysis demonstrated that the relationship between CT- and fluorescence-derived distribution volumes remained robust across all subsets of the data. The resulting *r*^2^ values ranged from 0.65 to 0.98, with a mean of 0.87, indicating that the correlation was not driven by any single data point.

In addition to the difference in distribution volume, there are notable differences in the concentration as a function of distance from the injection site. Plots of the relative concentration of fluorescent albumin and iodixanol as a function of distance from the centroid for 1-, 2-, and 4-mL injections are compiled in Fig. 4B. Relative concentration was normalized by dividing the measured injectate concentration in tissue by the concentration of the solution loaded into the syringe (i.e., the injection solution). Fluorescent albumin fluorescence intensity exhibits a slight radial dependence, which can be seen more clearly in the plots of individual samples in Fig. S1. At the centroid of the injectate distribution in tissue, fluorescent albumin was more concentrated than the injection solution (i.e., the relative concentration is greater than 1). The concentration dropped sharply at the boundary of the injectate distribution. In contrast, iodixanol dispersed through tissue with a maximum radiodensity at the centroid of approximately the same value as the injection solution, which decreases with distance. Individual samples are plotted in Fig. S2.

### Clinical implementation

**Fig. 5:**
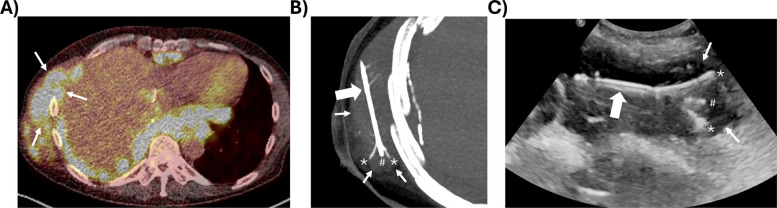
CT, US and PET of target tumor and multi-tine injection needle.** A** PET-CT fusion demarcates the tumor tissue (thin arrows) providing a target for drug delivery.** B** CT maximum intensity projection image verifies the position of the base needle (large arrow) and its tip (crosshatch) and 2 of 3 deployed injection tines (asterisks) within the tumor. Tines can be extended to enable 1 cm incremental deployments up to an array diameter up to 5 cm. To deliver drug to more tissue, the tines were partially retracted during injection. With full retraction, the needle could be rotated with redeployment and injection. **C** Ultrasound enabled real-time imaging of needle and tine location in relation to the fused US and CT and could also identify extravasation or extra-tumor leakage of the injectate

A dose fractionation strategy was employed with the total injection volume of 4 mL broken up into multiple low-volume injections through a multi-pronged needle with planned staged device repositioning to spread delivery through the visual tumor. The multi-pronged needle was composed of three injection needles that are advanced outward from a larger insertion needle to a maximum array diameter of 5 cm to distribute the injectate over multiple outputs. Tumor boundaries were defined on pre-procedural diagnostic imaging (Fig. 5A), and needle placement was verified with intra-procedural CT imaging (Fig. 5B). Needle repositioning and tine extension were guided by ultrasound imaging (Fig. 5C) as well as by ultrasound visualization of leakage or extravasation during injection.

## Discussion

Intratumoral injections have the potential to deliver highly concentrated and localized drug with reduced systemic exposure. However, intratumoral injections are more heterogenous than systemic administration because pressurized fluid may fracture tissue at the needle tip resulting in leakage of drug away from the injection site. The objective of this study was to determine the utility of co-injecting CT contrast with a fluorescently labeled surrogate drug to estimate drug delivery following direct tissue injection by comparing the distributions of the CT contrast with the surrogate drug. Intratumoral injections present logistical and technical challenges because there is a large parameter space to consider without consensus for technical guidelines or optimal intratumoral injection techniques [16]. The translational potential of this benchtop technique was illustrated by demonstrating that dividing the total injection volume into multifocal small injections enhances the localization of an injected drug, which then informed the injection technique implemented in a clinical study. Three-dimensional visualization of the distribution of an injected drug in tissue may facilitate a broader understanding of transport behavior and the interrelationship of injectate physical properties, injection parameters, and spatiotemporal concentration profiles that is integral to optimizing injection technique and generating technical guidelines.

In this study, the CT contrast agent iodixanol was co-injected with fluorescent albumin as a surrogate drug. Fluorescent albumin was selected as the surrogate drug because it is similar in size to LMB-100, an antibody-toxin conjugate undergoing clinical investigation [8]. The distribution volumes of the contrast agent and the estimate for the surrogate drug were different, but correlated (*R*^2^ = 0.89, Fig. 4A). The surrogate drug and CT imageable contrast have different transport behavior as evidenced by their respective radial concentration profiles in injected tissue that may be attributable to differences in hydrodynamic radius and zeta potential. Characterization of the hydrodynamic diameter and zeta potential at a neutral pH demonstrated that fluorescent albumin was larger (17.0 vs 2.7 nm) and more negatively charged (− 33 vs − 2.1 mV) than iodixanol (Fig. 1). In a dorsal skin fold window chamber model, fluorescent dextran of similar radius to fluorescent albumin, the surrogate drug in this study, exhibited shallower tumor penetration than dextran of similar radius to iodixanol following intravenous administration [17]. Iodixanol’s larger distribution volume and radial concentration dependence may be attributable to its smaller size and nearly neutral surface charge, which may allow it to permeate through the interstitial space. For iodixanol, the concentration was highest at the center of the injectate distribution, where it was equivalent to the concentration injected, and decreased towards the periphery (Fig. 2) with a more gradual slope indicative of diffusion [7].

Fluorescent albumin had a larger hydrodynamic radius that may restrict movement between cells and the extracellular matrix. Further, at a neutral pH, fluorescent albumin was above its isoelectric point and was negatively charged [18] which may have caused it to bind with collagen and positively charged proteins. These effects may have led to the diminished distribution volume with a uniform concentration profile and a steeper slope at the periphery compared to iodixanol (Fig. 3). Other drugs that are larger (e.g., larger antibodies, oncolytic viruses, nanoparticles) or more charged (e.g., doxorubicin) may lead to greater deviations in distribution as compared to iodinated contrast.

Intratumoral injections generate fluid pressures that deform tissue adjacent to the needle or force the injectate to flow through paths of least resistance. This process may lead to tissue fracture, leakage away from the injection site, and ultimately an unpredictable, non-uniform drug distribution [7]. Fractionating the total volume over several injections at different locations may mitigate leakage and may yield more localized delivery by minimizing local fluid pressure. Needles with multiple holes or deployable tines for drug delivery may achieve a similar effect. In this study, the normalized distribution volumes as measured with CT (Fig. 2F) and fluorescence imaging (Fig. 3F) illustrate that low-volume injections (i.e., 1 mL) had less variability than high-volume injections (i.e., 4 mL). Clinically, dose fractionation can be achieved with a multi-pronged needle that can be retracted, rotated, and repositioned to distribute drug throughout a large target tumor (Fig. 5), or by repeated repositioning of a single end-hole needle to achieve discrete low-volume injections.

The use of slow intratumoral injections to treat glioblastoma has achieved highly localized delivery with minimal leakage with a single injection [6, 19], but these procedures employ infusion rates around 1 µL/min and last for 24 h [6, 19], limiting more widespread application. Previous studies have established that infusion rates of 300 µL/min (0.3 mL/min) may fracture tissue, resulting in leakage of the injectate [20]. Our analysis demonstrates that dose fractionation may minimize leakage at clinically relevant infusion rates. The incorporation of a “fanning” technique (i.e., the needle is withdrawn during injection and then redirected and reinserted to create a fan shape[21]) has been implemented in the treatment of advanced melanomas with the oncolytic virus T-VEC [12], but no consensus exists regarding optimized injection strategies. A rationale for the broad application of a single injection strategy without consideration of drug physicochemical properties and heterogeneity of tumor biophysical properties is unlikely. The fractionation strategy described here may inform or inspire further study of similar paradigms with imageable drug surrogates or contrast to inform pre-procedural planning, needle selection and placement, infusion rate and volume, drug concentration, or the use of gelling polymers [22, 23].

There were limitations to this study. Due to background fluorescence introduced by bilirubin in liver tissue and quenching at high fluorescent concentrations, only a semi-quantitative assessment of the fluorescent albumin concentration was possible. To eliminate background fluorescence, a threshold equivalent to 0.2 mg/mL fluorescent albumin was implemented. As illustrated in Fig. 4B, the fluorescent intensity of the injectate exceeded 20 mg/mL, indicating that the injectate distribution volume measurements were not sensitive to the threshold selection.

Saturation was observed at concentrations equivalent to 200 mg/mL (Fig. 1), which was greater than concentrations observed experimentally. Fluorescence images were only acquired of sections near the center of the injectate, and this was used to approximate the distribution volume of the injectate by assuming a spherical distribution. Further, the calculated distribution volume measured with CT was dependent on the selected threshold value used for segmentation of the injectate due to the gradual decline in pixel intensity at the boundary between the injectate and surrounding tissue, as well as the limits of detection of iodine at low concentrations. Two different imaging modalities with differing spatial resolution were used to independently visualize the two components of the injectate. Estimation of the surrogate drug concentration profile from fluorescence imaging was subject to potential irregularities due to ice crystal formation from the slow freezing of large tissue volumes. Finally, these experiments were conducted in ex vivo normal liver tissue without recapitulating fluid flow and pressure gradients that exist in tumors in vivo. These deviations from the clinical scenario may result in an overestimation of the injectate distribution volume due to the absence of lymphatic and vascular clearance. Further, the lower interstitial fluid pressure of ex vivo tissue leads to a larger pressure gradient, which may also lead to larger distribution volume. Nevertheless, this approach allowed for a controlled evaluation of injection parameter effects on injectate distribution without confounding variables inherent to in vivo analyses.

## Conclusions

Intratumoral injections are an appealing alternative to systemic delivery with the potential to localize delivery of highly concentrated drug while reducing systemic exposure. This study demonstrated the potential utility of co-injection of a contrast agent to estimate the tissue distribution of a drug. The distributions of contrast agent and a model surrogate drug differed due to the limited diffusion of the larger and negatively charged surrogate drug but were spatially correlated. Smaller injection volumes resulted in less variable tissue distribution and were less prone to leakage of the injectate from tissue compared to larger volume injections. This finding suggests that fractionation of the total injection volume over multiple injections may benefit on-target drug delivery. Multi-focal low-volume injections may be preferable and result in more predictable delivery, such as with the use of a multi-pronged needle or repeated small multi-focal injections from a single end-hole needle.

## Supplementary Information

Supplementary Material 1: Figure S1. Compiled fluorescence intensity curves as a function of radial distance for 1 mL (A), 2 mL (B), and 4 mL (C) injections. Dotted lines represent individual trials and solid lines represent the average. Only 2 samples are plotted for the 4 mL injections (C) because 1 sample was excluded due to lack of fluorescent signal. Concentrations were estimated from calibration standards in Fig. 1.

Supplementary Material 2: Figure S2. Compiled radiodensity curves as a function of radial distance for 1 mL (A), 2 mL (B), and 4 mL (C) injections. Dotted lines represent individual trials and solid lines represent the average.

## Data Availability

The datasets generated during and/or analyzed during the current study are available from the corresponding author on reasonable request.
