# Supplementary material for: Mapping drug distribution using CT imaging following direct tissue injection in ex vivo liver: informing clinical implementation
Source: CVIR Oncol. 2025 Dec 16;1(1):27. doi: 10.1007/s44343-025-00027-x (PMC12716157; doi:10.1007/s44343-025-00027-x)
Supplement: Supplementary file 1 — Supplementary Material 1: Figure S1. Compiled fluorescence intensity curves as a function of radial distance for 1 mL (A), 2 mL (B), and 4 mL (C) injections. Dotted lines represent individual trials and solid lines represent the average. Only 2 samples are plotted for the 4 mL injections (C) because 1 sample was excluded due to lack of fluorescent signal. Concentrations were estimated from calibration standards in Fig. 1. [file 44343_2025_27_MOESM1_ESM.docx]

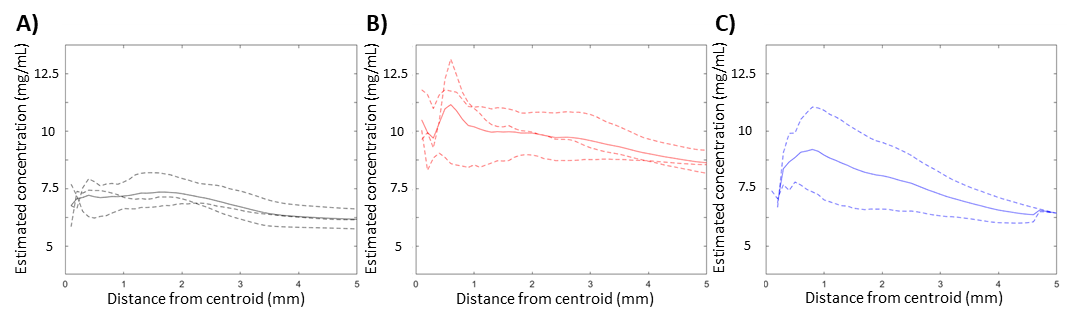


**Supplementary Figure 1:** Compiled fluorescence intensity curves as a function of radial distance for 1 mL (**A**), 2 mL (**B**), and 4 mL (**C**) injections. Dotted lines represent individual trials and solid lines represent the average. Only 2 samples are plotted for the 4 mL injections (**C**) because 1 sample was excluded due to lack of fluorescent signal. Concentrations were estimated from calibration standards in **Fig. 1**.
