# Supplementary material for: Mapping drug distribution using CT imaging following direct tissue injection in ex vivo liver: informing clinical implementation
Source: CVIR Oncol. 2025 Dec 16;1(1):27. doi: 10.1007/s44343-025-00027-x (PMC12716157; doi:10.1007/s44343-025-00027-x)
Supplement: Supplementary file 2 — Supplementary Material 2: Figure S2. Compiled radiodensity curves as a function of radial distance for 1 mL (A), 2 mL (B), and 4 mL (C) injections. Dotted lines represent individual trials and solid lines represent the average. [file 44343_2025_27_MOESM2_ESM.docx]

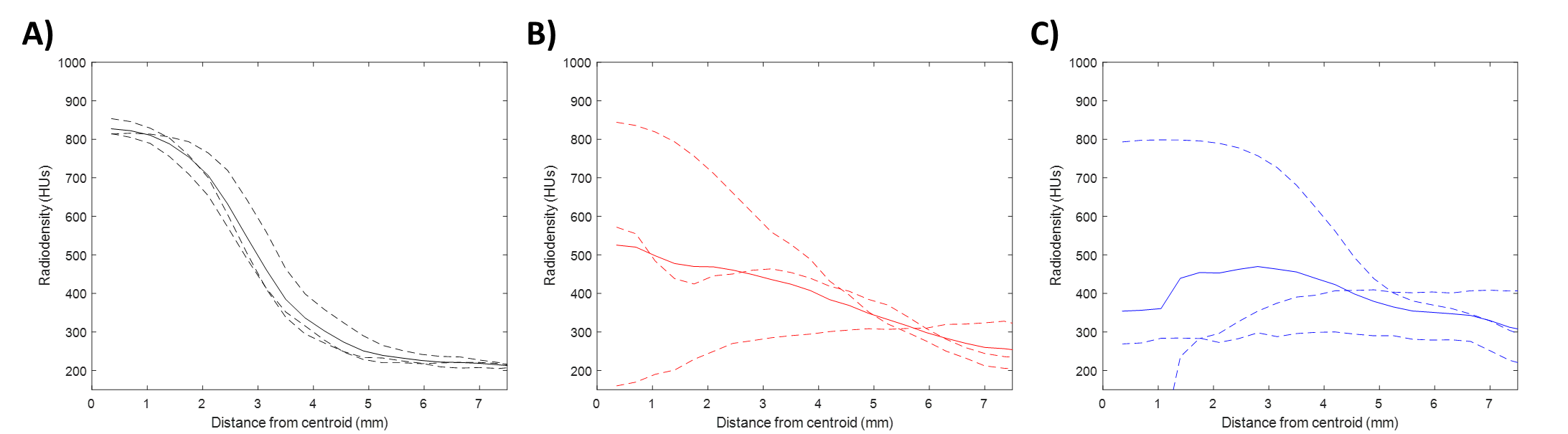


**Supplementary Figure 2:** Compiled radiodensity curves as a function of radial distance for 1 mL (**A**), 2 mL (**B**), and 4 mL (**C**) injections. Dotted lines represent individual trials and solid lines represent the average.
